# Fingolimod Modulates the Gene Expression of Proteins Engaged in Inflammation and Amyloid-Beta Metabolism and Improves Exploratory and Anxiety-Like Behavior in Obese Mice

**DOI:** 10.1007/s13311-023-01403-2

**Published:** 2023-07-11

**Authors:** P. L. Wencel, K. Blecharz-Klin, A. Piechal, J. Pyrzanowska, D. Mirowska-Guzel, R. P. Strosznajder

**Affiliations:** 1 Laboratory of Preclinical Research and Environmental Agents, Mossakowski Medical Research Institute, Polish Academy of Sciences, 5 Pawinskiego St., 02106 Warsaw, Poland; 2 Department of Experimental and Clinical Pharmacology, Centre for Preclinical Research and Technology CePT, Medical University of Warsaw, 1B Banacha St., 02097 Warsaw, Poland

**Keywords:** Obesity, Sphingolipids, Sphingosine-1-phosphate receptor modulator, Fingolimod, Inflammation, Behavior

## Abstract

Obesity is considered a risk factor for type 2 diabetes mellitus, which has become one of the most important health problems, and is also linked with memory and executive function decline. Sphingosine-1-phosphate (S1P) is a bioactive sphingolipid that regulates cell death/survival and the inflammatory response via its specific receptors (S1PRs). Since the role of S1P and S1PRs in obesity is rather obscure, we examined the effect of fingolimod (an S1PR modulator) on the expression profile of genes encoding S1PRs, sphingosine kinase 1 (*Sphk1*), proteins engaged in amyloid-beta (Aβ) generation (ADAM10, BACE1, PSEN2), GSK3β, proapoptotic Bax, and proinflammatory cytokines in the cortex and hippocampus of obese/prediabetic mouse brains. In addition, we observed behavioral changes. Our results revealed significantly elevated mRNA levels of *Bace1*, *Psen2*, *Gsk3b*, *Sphk1*, *Bax*, and proinflammatory cytokines, which were accompanied by downregulation of *S1pr1* and sirtuin 1 in obese mice. Moreover, locomotor activity, spatially guided exploratory behavior, and object recognition were impaired. Simultaneously, fingolimod reversed alterations in the expressions of the cytokines, *Bace1*, *Psen2*, and *Gsk3b* that occurred in the brain, elevated *S1pr3* mRNA levels, restored normal cognition-related behavior patterns, and exerted anxiolytic effects. The improvement in episodic and recognition memory observed in this animal model of obesity may suggest a beneficial effect of fingolimod on central nervous system function.

## Introduction

Obesity and type 2 diabetes mellitus (T2DM) is a fast-growing worldwide epidemic that has become one of the most common health problems in many industrialized countries and inevitably lowers the quality of life on social and economic levels. Data from epidemiological studies demonstrate that in 2019, over 463 million people were diagnosed with diabetes, and 4.2 million died because of diabetes. Scientists estimate that in 2030, there will be approximately 578 million people with diabetes, with a 10% global prevalence [1]. The consumption of a high-fat diet (HFD) and lack of physical activity are major factors contributing to obesity and its comorbidities, and more than 80% of obese patients are believed to have T2DM [2]. A recently published neuroimaging meta-analysis identified many spontaneous abnormal central nervous system (CNS) activities in T2DM patients, mainly involving brain regions related to memory, learning, and emotions [3]. Functional deficits of the CNS and structural brain damage in regions involved in cognition, anxiety, and depression control were observed in T2DM patients [4]. Dysregulation of insulin signaling observed in obesity, metabolic syndrome, and T2DM extends to the brain and can be responsible for a broad spectrum of brain disorders. A comprehensive cohort study of nearly 1.9 million adults presented by Wimberley et al. in 2022 confirms that T2DM is correlated with a wide range of psychiatric and neurological problems, including depression, obsessive–compulsive disorder, inflammatory brain diseases, vascular dementia, and cognitive impairment [5]. Obesity has also been associated with a higher risk of developing cognitive disorders, which include dementia and its most common form, Alzheimer’s disease (AD) [6–8]. Unfortunately, the role of obesity in the etiology of deficits in executive function and memory is still not well understood. Some research suggests that obesity-associated chronic inflammation comes from altered physiology of adipose tissue (AT), resulting in low-systemic insulin resistance and metabolic dysfunction that, in consequence, may lead to brain dysfunction [9, 10]. Studies conducted on an experimental mouse model of senescence revealed that HFD consumption increased cerebral amyloid-beta peptide (Aβ) accumulation, deregulated tau-phosphorylating glycogen synthase kinase 3β (GSK3β) immunoreactivity, and aggravated learning and memory deficits, indicating Alzheimer-like changes [11]. Continuous HFD consumption may result in obese conditions that lead to increased AT lipolysis, plasma free fatty acids, and finally to the accumulation of bioactive lipids, including ceramide (Cer), which promotes lipotoxicity [12]. Bioactive sphingolipids are a lipid family that regulates cellular signaling and function. There is particular interest in them as a HFD increases their levels, changes energy metabolism, and importantly they contribute to obesity-related pathologies. Among the members of the bioactive sphingolipid family, Cer and sphingosine-1-phosphate (S1P) are the most important because of their regulatory function in cells. Both Cer and S1P, as well as products of their metabolism, regulate a vast array of biological processes, from programmed cell death and senescence to cell survival and proliferation, whereas Cer is mostly recognized for the regulation of apoptotic processes [13, 14]. Cer and S1P are also important regulators of inflammatory signaling. S1P is synthetized/generated through phosphorylation of sphingosine (a product of Cer metabolism) by sphingosine kinases (SPHKs). S1P regulatory functions are mediated by five G protein-coupled S1P receptor (S1PR) subtypes. Different receptor expression and specificity of S1P toward G-proteins explain other but overlapping roles of S1PRs. The vast functions of S1P and its receptors have become potential pharmacological targets for the treatment of inflammation-related disorders. FTY720 is an orally bioavailable structural analog of sphingosine that can be phosphorylated by SPHKs to FTY720 phosphate and act as a S1PRs modulator. FTY720 modulates four (1, 3, 4, and 5) out of five S1P receptor subtypes. FTY720, also known as fingolimod, is able to cross the blood–brain barrier due to its lipophilic nature and is approved for the treatment of the relapsing–remitting form of multiple sclerosis (MS) [15, 16]. In addition to its immunomodulatory activity, fingolimod is also a potent modulator of genes involved in ceramide metabolism, and it stimulates neuronal gene expression, axonal growth, and regeneration during neurodegeneration [17, 18]. Our previous results revealed that FTY720 recovers the altered expression of genes encoding SPHKs, S1P receptor 1 and 3, ceramide kinase, and BCL-2 protein in the brains of AD mice [19]. In the case of diabetes, it was found that FTY720 administration led to the normalization of hyperglycemia by stimulating β-cell regeneration in vivo and regulating circulating insulin levels without affecting insulin sensitivity [20]. Moreover, FTY720 was found to reverse HFD-induced weight gain in C57BL/6 mice as well as insulin resistance and AT inflammation [21].

Recently, the role of bioactive sphingolipids and their signaling in glucose homeostasis, insulin signaling, and diabetic phenotype started to draw attention, primarily because of evidence linking ceramides to insulin resistance [22]. While the role of ceramides in obesity and diabetes is relatively well understood, the role of S1P and its receptors is rather unclear. Elevated plasma levels of S1P have been observed in animal models of obesity and T2DM as well as in obese patients [23, 24]. S1P is an important regulator of glucose-stimulated insulin secretion in pancreatic beta cells. Some data show that receptors for S1P are engaged in the regulation of inflammatory signaling, specifically receptors 2 and 3, but in the case of obesity and diabetes, S1P-S1PR3 signaling seems to have a protective function [25, 26].

Since the role of SphK1/S1P and S1P receptors in obesity is still not well understood, we investigated the role of S1P receptors and the effect of fingolimod on the expression of several genes encoding sphingosine kinase 1, S1P receptors, ADAM10, BACE1, PSEN2, GSK3β, SIRT1, proapoptotic Bax, and proinflammatory cytokines in the brains of obese mice. Moreover, behavioral changes in animals receiving a HFD as well as the response to the fingolimod were assessed.

## Materials and Methods

### Animal Model and Treatment

**Fig. 1:**
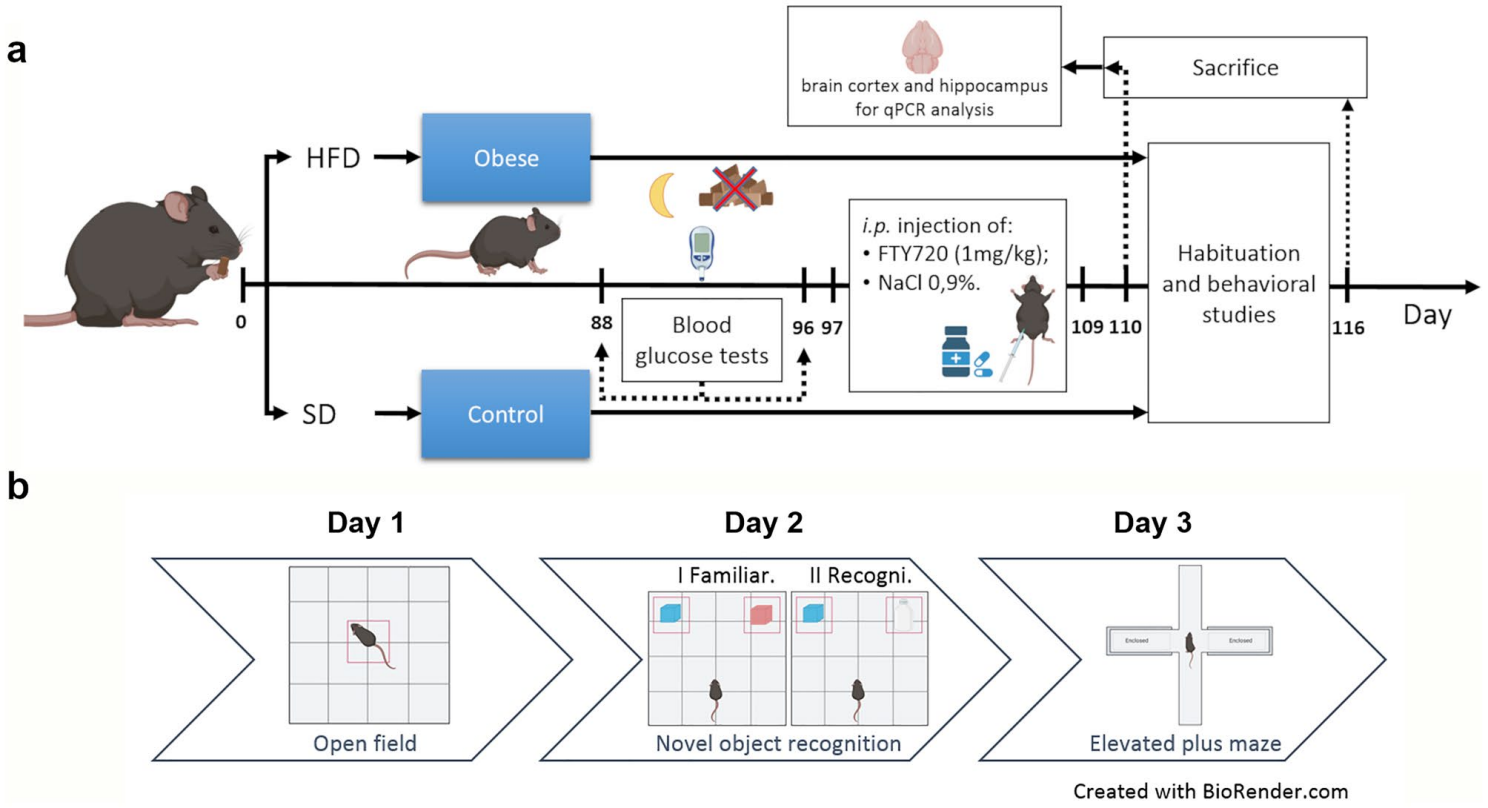
Schematic representation of the obese mouse model (**a**) and behavioral tests (**b**) used in this study (created with Biorender.com)

All experiments were approved by the II Local Ethics Committee for Animal Experimentation in Warsaw (approval no. WAW2/065/2019) and performed in accordance with the guidelines of EU Directive 2010/63/EU. Male C57BL/6 J mice (10–12 weeks, 27 ± 2 g) from The Animal House of the Mossakowski Medical Research Institute PAS, Warsaw, Poland, were given a HFD (Ssniff: 60 kJ% fat, 20 kJ% protein, 20 kJ% carbohydrates, E15742-34, corresponding to Research Diets, Inc. D12492) for 16 weeks. The control group received a standard chow diet (SD, Ssniff: 9 kJ% fat, 24 kJ% protein, 67 kJ% carbohydrates, S8435). All groups of mice were housed individually in plastic breeding cages in an air-conditioned room with mechanical ventilation, a regulated 12-h dark–light cycle, a temperature of 20–24 °C, and humidity of 55% (± 10%). In all cages, environmental enrichment in the form of cotton rolls and wooden fibers for building a nest was applied. At the beginning of the experiment, no statistically significant body weight differences existed between the experimental groups. On a 12-week HFD, blood glucose tests were performed after an overnight (14–16 h) fast. After 96 days on a HFD, animals started receiving FTY720 (i.p. 1 mg/kg b.w.; Cayman Chemical) in 0.9% NaCl once daily for 2 weeks; control animals received vehicle (NaCl solution). One day after the last FTY720 injection, the animals were sacrificed by decapitation. The cortex and hippocampus were quickly isolated on an ice-cold glass Petri dish, and samples were immediately frozen in liquid nitrogen and stored at − 80 °C for qPCR analysis. Twenty animals were used for the gene expression study, and 28 animals were used for the behavioral studies. Behavioral experiments started 4 days after the last injection of FTY720 with an open field test and were conducted from 9:00 to 16:00 for three consecutive days (Fig. 1a, b). The behavioral studies were performed blinded and carried out in compliance with the ARRIVE guidelines (https://arriveguidelines.org). The behavior of the animal was scored by observers blinded to the experimental plan and experimental conditions. Animal behavior was monitored with a video camera located above the behavioral apparatus and recorded with Noldus EthoVision XT10 software. Behavioral data were analyzed by other researchers not involved in the study design. The resting time between particular behavioral tests was 24 h. To acclimate, mice were moved from a housing room to an experimental room where the behavioral tests were performed for at least 1 h prior to use each day. Mice in the present study were handled frequently prior to the behavioral test (1–2 times/day for at least 1 min during the 2 weeks prior to testing).

### Behavioral Tests

#### Open Field Test

The open field test (OF) was originally developed by Hall to monitor motor activity and the degree of exploration in a new place [27]. In the OF test, we used naive animals to reduce habituation to the test environment and eliminate any potential crossover effect from multiple apparatus exposures. The mice were placed in an open gray area (57 × 57 cm) bounded by walls measuring 50 cm with an open top. The OF apparatus was located in an acoustically isolated experimental room illuminated by diffused light. The mouse was placed individually in the center of the OF arena for 8 min. The OF test involved measurements of parameters such as climbing, grooming, total distance traveled, velocity, and time spent in the central segments of the box. Due to the high sensitivity of the test, it was conducted without the presence of an observer in the experimental room. After 8 min, the animal was removed, and the apparatus was washed with a 10% ethanol solution to remove odor traces. The box used for OF was also used during the novel object recognition test performed the next day.

#### Novel Object Recognition Test

The novel object recognition (NOR) test has been primarily used in rats and was originally described by Ennaceur and Delacour in 1988 and then successfully adapted for mice [28–31]. The NOR test is a commonly used, relatively low-stress behavioral assay for investigating various aspects of learning and memory in rodents and an efficient means for evaluating various stages of memory, for example, acquisition, consolidation, and recall [32]. NOR allows for an assessment of episodic memory and deficits in recognition memory which lead to the impaired ability to discriminate between a new and a familiar object [33, 34], and helps in the assessment of neuropsychological changes [32]. In the NOR test, object location memory (spatial object memory) can also be investigated (e.g., [35]). During the task, memory is consolidated, and spatial/contextual characteristics of objects are relocated in different parts of the brain [36]. The task procedure was conducted in the previously described OF apparatus and consisted of three phases: habituation, familiarization, and choice. Based on our optimization of experimental conditions, the first phase, habituation to the apparatus, was carried out during the OF test, where each animal was allowed to freely explore the open arena for 8 min in the absence of objects. During the familiarization phase on the second day, the animals were placed against the wall opposite two identical objects A1 and A2 (Lego towers) located at opposite corners of the apparatus. The animals were allowed to explore the apparatus for 8 min freely. After this time, the mice were removed from the box, and the apparatus and objects were cleaned with a 10% ethanol solution to remove odor cues. Two hours after the familiarization phase, one of the two objects was replaced with another/novel object (object B—glass bottle), and the choice phase began. New and familiar objects came in different colors, shapes, and structures. Object exploration was classified as placement of the mouse head within 2 cm of any object. Each mouse was placed near the center of the wall opposite the object in the same box to explore the arena for 8 min. The activity of the exploring animal was monitored with a camera located above the apparatus and recorded with a computer program. The time spent exploring individual objects during the familiarization (*t*_A1_, *t*_A2_) and choice phases (*t*_A1_, *t*_B_), total time spent exploring both objects during the familiarization (*t*_A1A2_) and choice phases (*t*_A1B_), total time of exploration [*t*_total_ = (*t*_A1A2_ + *t*_A1B_)], discrimination index for familiarization phase − exploration time devoted to both identical objects [DI_A1A2_ = (*t*_A1_ − *t*_A2_)/(*t*_A1_ + *t*_A2_)], and discrimination index for the choice phase − the difference in exploration time for the novel versus familiar object [DI_A1B_ = (*t*_B_ − *t*_A1_)/(*t*_B_ + *t*_A1_)] were all calculated. The global habituation index was also determined by comparing the total time spent exploring the two objects during the familiarization phase to that spent during the choice phase [GHI = *t*_A1A2_/*t*_A1B_]. The recognition index [RI = *t*_B_/*t*_total_], defined as the time spent investigating the novel object relative to the total object exploration, was also measured.

$${DI}_{A1A2}= \frac{{t}_{A1}-{t}_{A2}}{{t}_{A1}+{t}_{A2}} ,\quad {DI}_{A1B}= \frac{{t}_{B}-{t}_{A1}}{{t}_{B}+{t}_{A1}} ,\quad GHI= \frac{{t}_{A1A2}}{{t}_{A1B}},\quad RI= \frac{{t}_{B}}{ {t}_{total}}$$

#### Elevated Plus Maze Test

Following the OF and NOR tests, a counterbalanced test of anxiety and exploration was conducted on the third day. The elevated plus maze (EPM) test is used to measure anxiety-related behavior based on the natural, spontaneous exploratory behavior of rodents in novel environments as well as their aversion to open and elevated spaces [37]. The EPM consisted of four narrow gray arms (31 cm × 6.5 cm × 16 cm, L × W × H) placed at a height of approximately 50 cm. The two arms facing each other were enclosed by gray sidewalls (closed arms) and were connected via a central square to two open arms. The test was carried out in an acoustically insulated experimental room illuminated by diffused light. The mouse was placed in the maze at the crossing of the arms, with its nose pointing toward the open arm. The activity of the exploring animal was monitored for 8 min. After this time, the animal was taken from the EPM. Then, the maze was cleaned with a 10% ethanol solution.

#### Intraperitoneal Glucose Tolerance Test

Glucose levels were measured and an intraperitoneal glucose tolerance test (IPGTT) was performed in mice after 12 weeks on a HFD. Animals fasted overnight for 16 h (6:00 pm–10:00 am) before the glucose tolerance test. The animals were injected with glucose (2 g/kg b.w./i.p., dissolved in saline). Blood glucose levels were measured from the tail vein with an Accu-Check Performa glucometer (Roche) immediately prior to glucose injection (0 min) and at 15, 30, 60, and 120 min after glucose administration.

### Gene Expression Analysis

The cortex and hippocampus were isolated on ice and flash-frozen in liquid nitrogen. RNA was extracted using TRI reagent according to the manufacturer’s protocols (Sigma‒Aldrich/Merck). DNA was digested with DNase I (Sigma‒Aldrich/Merck). The concentration and purity of RNA were assessed spectrophotometrically (A260/A280 method). Reverse transcription of 4 μg of total RNA was performed with avian myeloblastosis virus reverse transcriptase and random primers (High Capacity Reverse Transcription Kit; Applied Biosystems). Real-time PCR was performed with TaqMan Gene Expression Assay kits on an ABI PRISM 7500 machine (both reagents and equipment—Applied Biosystems) using specific mouse primers: *Sphk1* (Mm00448841_g1), *S1pr1* (Mm02619656_s1), *S1pr3* (Mm02620181_s1), *Il1b* (Mm01336189_m1), *Il6* (Mm00446190_m1), *Tnf* (Mm00443258_m1), *Sirt1* (Mm01168521_m1), *Bax* (Mm00432051_m1), *Adam10* (Mm00545742_m1), *Bace1* (Mm00478664_m1), *Psen2* (Mm00448413_m1), and *Gsk3b* (Mm00444911_m1). Each sample was analyzed in triplicate or quadruplicate. Gene expression was calculated using the ΔΔCt method and normalized against beta-actin (Actb, Mm00607939_s1).

### Statistical Analysis

The mRNA expression levels (RQ), blood glucose levels, and animal weight are presented as the means ± SEMs. Data, depending on experimental design, were analyzed using Student’s *t* test or analysis of variance (one- or two-way ANOVA) followed by a post hoc test. The behavioral data from the OF, EPM, and NOR tests were analyzed by one-way analysis of variance (ANOVA) with Tukey’s post hoc test for multiple comparisons. The statistical analyses were performed using GraphPad Prism 6 (GraphPad Software, San Diego, CA). Statistical significance was accepted at *p* < 0.05.

## Results

### Changes in Body Weight and Blood Glucose Levels

**Fig. 2:**
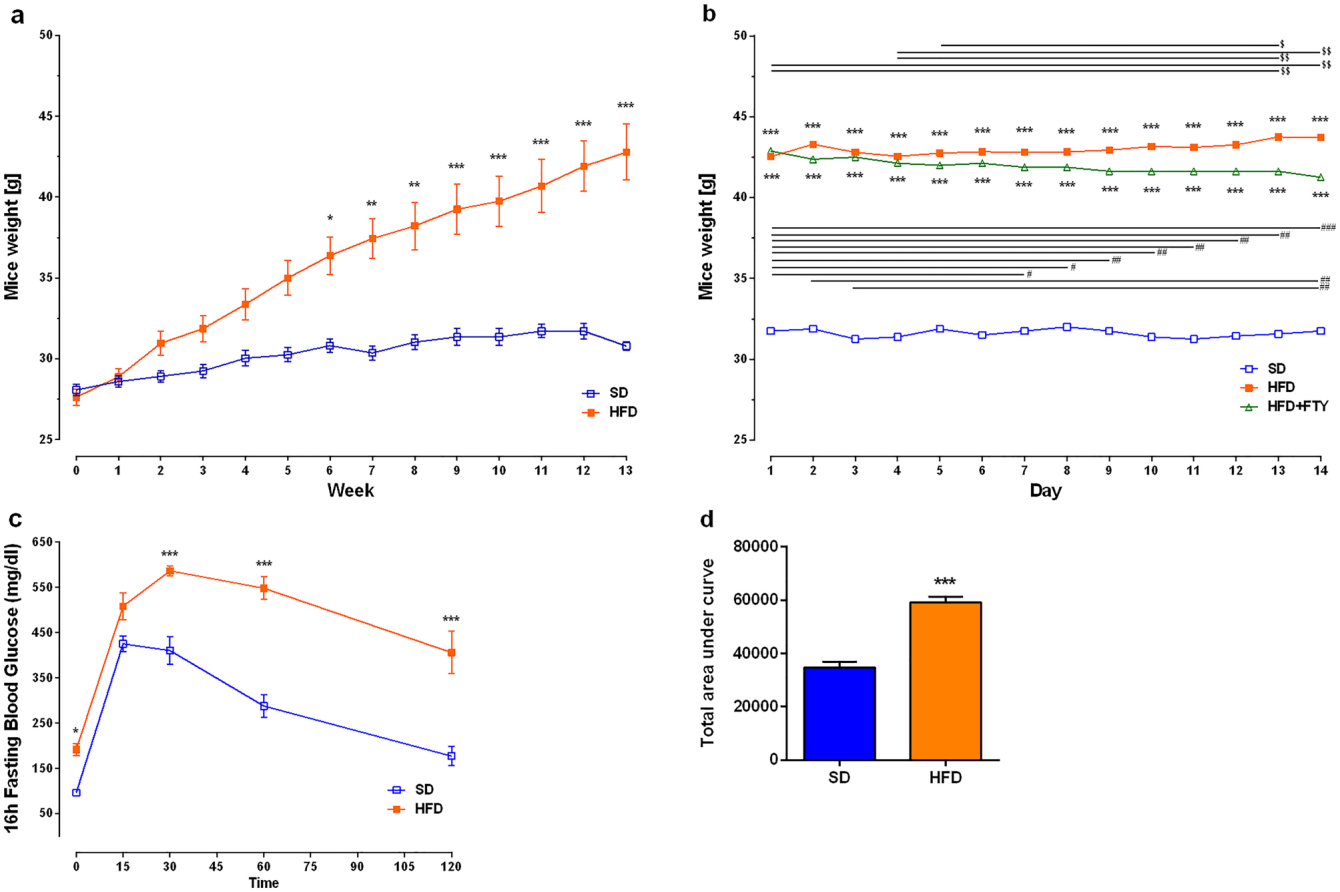
Effect of a HFD on animal body weight (g) and blood glucose levels. Animal weight from the beginning of the experiment until the glucose test (**a**, SD: *n* = 8, HFD: *n* = 16) and during administration of FTY720 (**b**, *n* = 8 per group); IPGTT (**c**) and AUC (**d**) after 16 h of fasting (SD: *n* = 7, HFD: *n* = 7). For statistical comparison, Student’s *t* test or two-way ANOVA with Sidak’s post hoc test was used. *P* values < 0.05 were considered statistically significant (*p < 0.05; **p < 0.01, ***p < 0.001 compared to the SD mice; ^#^*p* < 0.05; ^##^*p* < 0.01, ^###^*p* < 0.001 HFD + FTY animals mass compared to the different day of administration; ^$^*p* < 0.05; ^$$^*p* < 0.01 HFD animals mass compared to the different day of administration)

The weight of obese mice was significantly higher than that of SD mice starting at 6 weeks on a HFD (Fig. 2a). FTY720 significantly reduced the weight of HFD animals, while HFD mice receiving vehicle continued to gain weight (Fig. 2b). Fasting blood glucose levels were almost two times higher compared to the mice that were on a standard diet (Fig. 2c). During the IPGTT, blood glucose levels reached peak values at 15 min for SD mice and 15 min later for HFD mice and then began to decline, reaching 177 mg/dL for SD mice after 120 min and 406 mg/dL for HFD mice. These results were further supported by the areas under the curve (AUC) (Fig. 2d).

### Changes in Sphk1, S1P Receptors, and Proinflammatory Cytokine Gene Expression in the Cortex and Hippocampus of Obese Mouse Brains

**Fig. 3:**
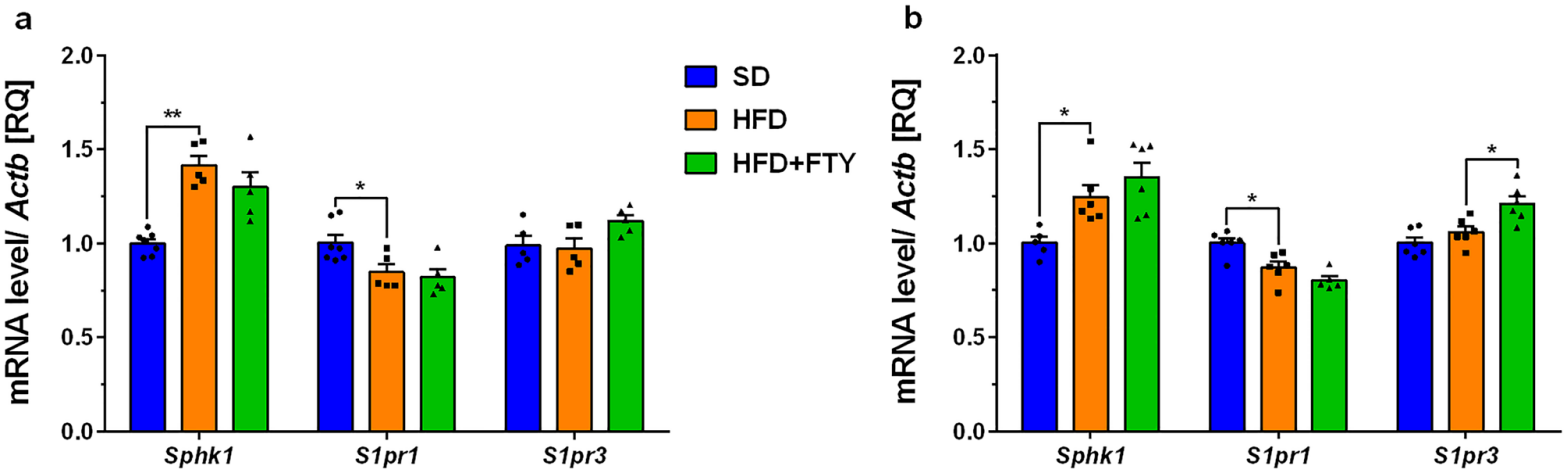
Changes in the mRNA levels of sphingosine kinase 1 (*Sphk1*) and receptors of sphingosine-1-phosphate (*S1pr1* and *3*) measured using real-time PCR in the brain: cortex (**a**) and hippocampus (**b**) of control animals on a standard diet (SD), animals receiving only a high-fat diet (HFD) and mice simultaneously treated with FTY720 (HFD + FTY). *p < 0.05; **p < 0.01 compared to the appropriate controls (*n* = 15–18 for each gene, 5–7 for each group); ANOVA with Tukey’s post hoc test

**Fig. 4:**
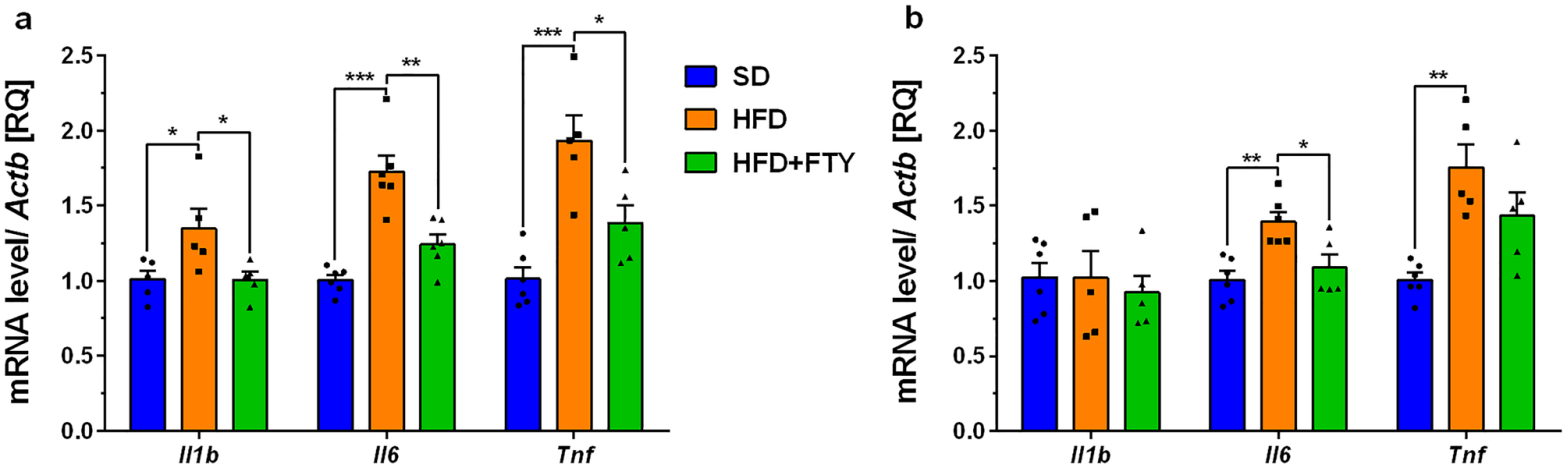
Changes in the mRNA levels of proinflammatory cytokines *Il1β*, *Il6*, and *Tnf* measured using real-time PCR in the cortex (**a**) and hippocampus (**b**) of standard diet (SD), high-fat diet (HFD), and HFD-fed mice administered FTY720 (HFD + FTY). *p < 0.05; **p < 0.01; ***p < 0.001 compared to the appropriate controls (*n* = 15–18 for each gene, *n* = 5–6 for each group); ANOVA with Tukey’s post hoc test

In the cortex of obese mouse brains, we observed significant upregulation of sphingosine kinase 1 (*Sphk1*) gene expression with concomitant reduction of sphingosine-1-phosphate receptor 1 (*S1pr1*) mRNA levels (Fig. 3a). Similar to the changes that we observed in the obese mouse cortex, the hippocampal expression of *Sphk1* was significantly elevated, which was also accompanied by the downregulation of *S1pr1* (Fig. 3b). The administration of FTY720 significantly upregulated the expression of *S1pr3* in the hippocampus (Fig. 3b). We also observed significant upregulation of genes encoding the proinflammatory cytokines interleukin 1b (*Il1b*), interleukin 6 (*Il6*), and tumor necrosis factor α (*Tnf*) in the cortex of mice consuming a HFD, which was followed by upregulation of *Il6* and *Tnf* in the hippocampus (Fig. 4a, b). Fingolimod significantly reversed changes in mRNA levels in the cortex and reduced *Il6* mRNA levels, with concomitant slight downregulation of *Tnf* gene expression in the hippocampus (Fig. 4a, b).

### Changes in APP Metabolism-Related Proteins, Gsk3β, and Bax Gene Expression in the Cortex and Hippocampus of Obese Mouse Brains

**Fig. 5:**
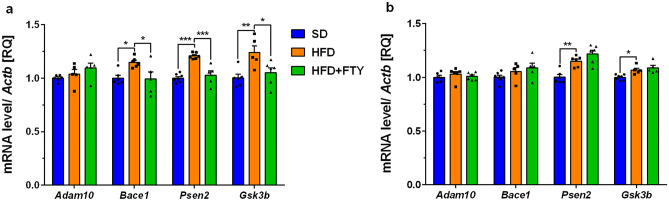
Changes in the mRNA levels of secretases (*Adam10, Bace1*, and subunit *Psen2*) and glycogen synthase kinase-3 beta (*Gsk3b*) measured using real-time PCR in the cortex (**a**) and hippocampus (**b**) of standard (SD), high-fat diet (HFD), and HFD mice administered FTY720 (HFD + FTY). *p < 0.05; **p < 0.01; ***p < 0.001 compared to the appropriate controls (*n* = 16–18 for each gene, *n* = 5–7 for each group); ANOVA with Tukey’s post hoc test

**Fig. 6:**
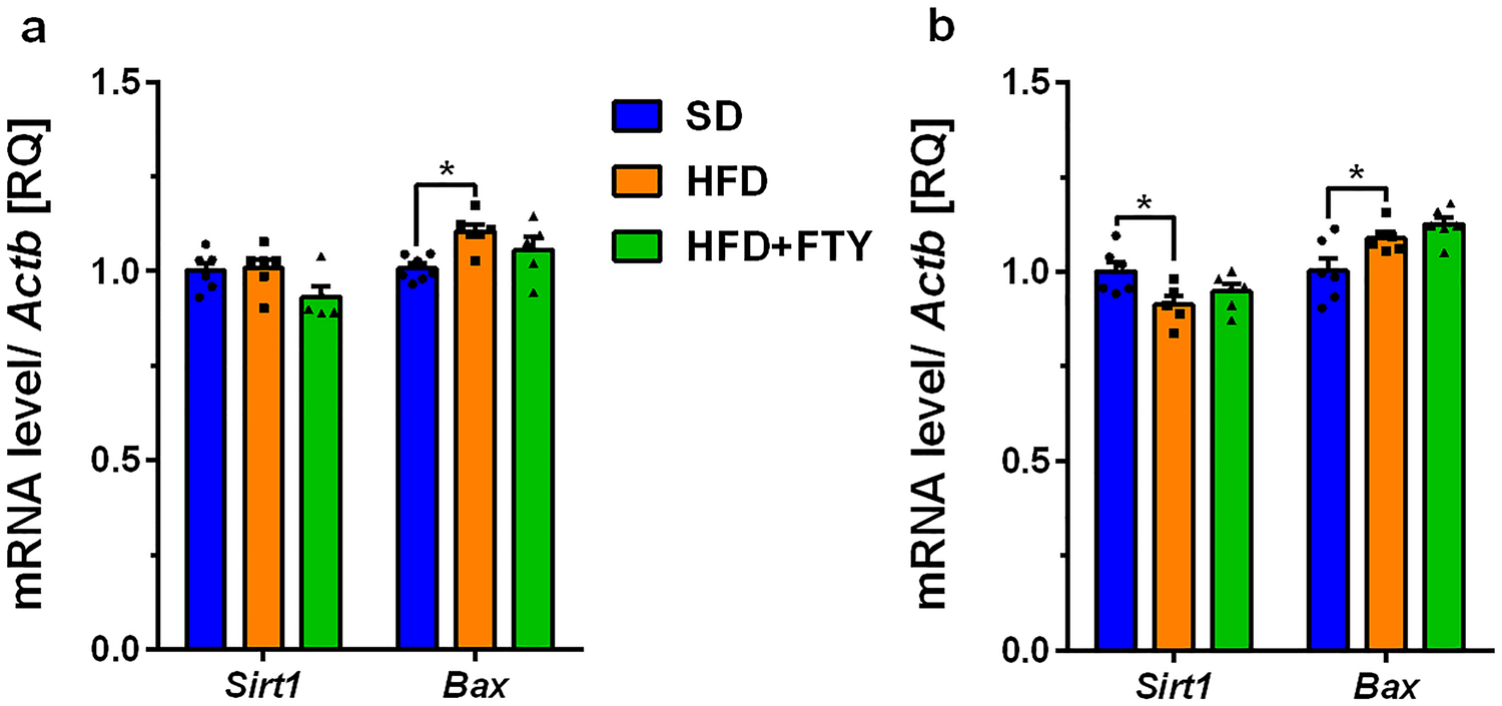
Changes in the mRNA levels of sirtuin 1 (*Sirt1*) and Bcl-2-associated X protein (*Bax*) measured using real-time PCR in the cortex (**a**) and hippocampus (**b**) of standard (SD), high-fat diet (HFD), and HFD mice administered FTY720 (HFD + FTY). *p < 0.05 compared to the appropriate controls (*n* = 16–18 for each gene, *n* = 5–7 for each group); ANOVA with Tukey’s post hoc test

The mean expression levels of genes encoding beta-secretase 1 (*Bace1*), presenilin 2 (*Psen2*), and glycogen synthase kinase-3 beta (*Gsk3b*) were significantly higher in the cortex of animals on a HFD compared to control animals (SD) (Fig. 5a). Only *Psen2* expression was significantly upregulated in the hippocampus of obese mice (Fig. 5b). Modulation of S1PRs by FTY720 significantly reversed all these changes exclusively in the cortex by returning elevated values to control levels (Fig. 5a). We also observed significantly increased cortical mRNA levels of *Bax*, which encodes the proapoptotic Bcl-2-associated X protein, in mice that were fed a HFD (Fig. 6a). Moreover, significant elevation of *Gsk3b* and pro-apoptotic *Bax* with accompanying reduction of pro-survival sirtuin 1 (*Sirt1*) gene expression was observed in the hippocampus of HFD mice (Figs. 5b, 6b).

### Behavioral Tests

#### Open Field Test

**Fig. 7:**
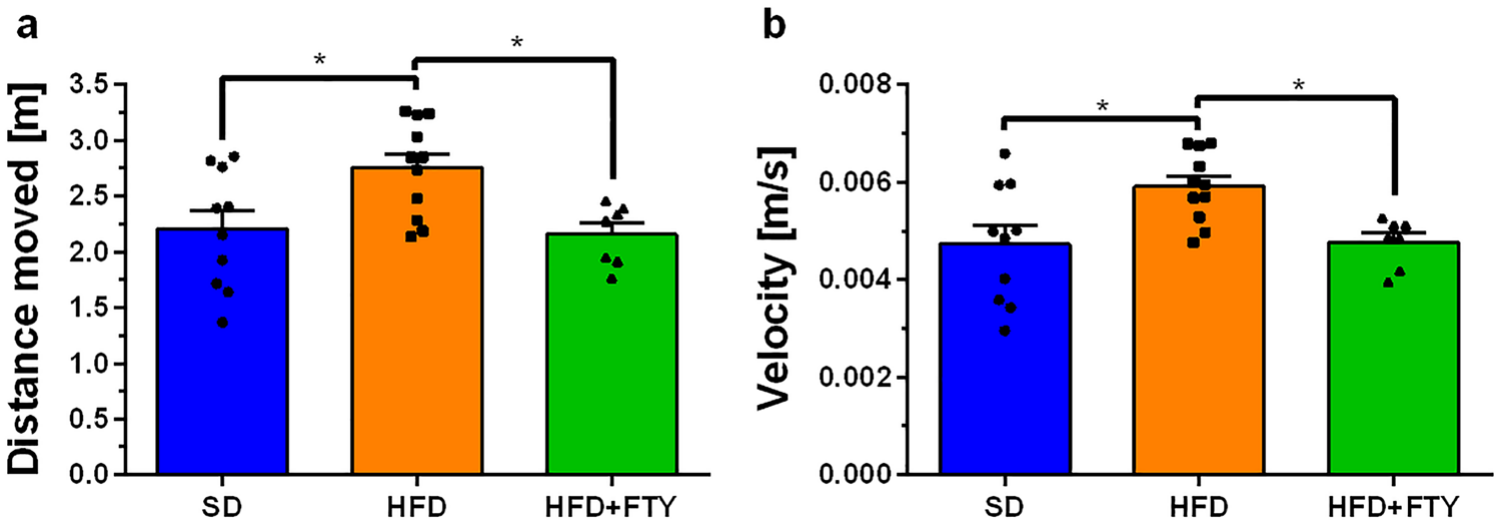
Main OF locomotor parameters for the mice that received a standard diet (SD, *n* = 10), mice fed a high-fat diet (HFD, *n* = 11), and animals treated with FTY720 (HFD + FTY, *n* = 7). Animals that received HFD showed hyperactivity and exhibited increased distance moved (**a**) and velocity (**b**), but administration of FTY720 reversed this effect. *HFD vs. SD, HFD + FTY, *p* < 0.05 (ANOVA with Tukey’s post hoc test)

**Fig. 8:**
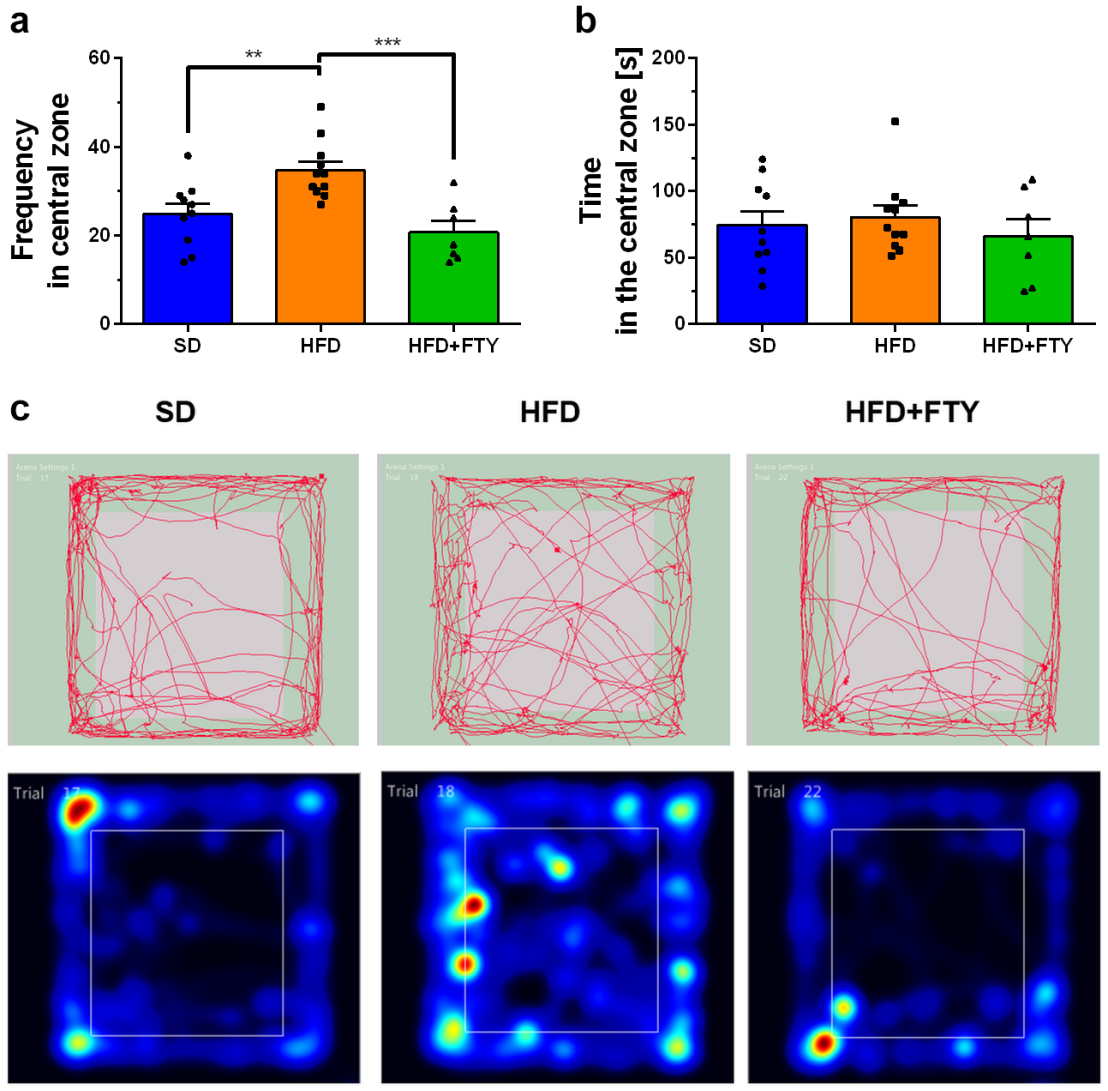
Frequency in the center zone of the OF apparatus (**a**), time spent in the central part of the testing box (**b**), and representative movement trajectory: track plot and heatmaps–occupancy plot (**c**) of mice that received a standard diet (SD, *n* = 10), mice fed a high-fat diet (HFD, *n* = 11), and animals additionally treated with FTY720 (HFD + FTY, *n* = 7). **HFD vs. SD, *p* < 0.01 (Tukey test); ***HFD vs. HFD + FTY, *p* < 0.001 (ANOVA with Tukey’s post hoc test)

**Fig. 9:**
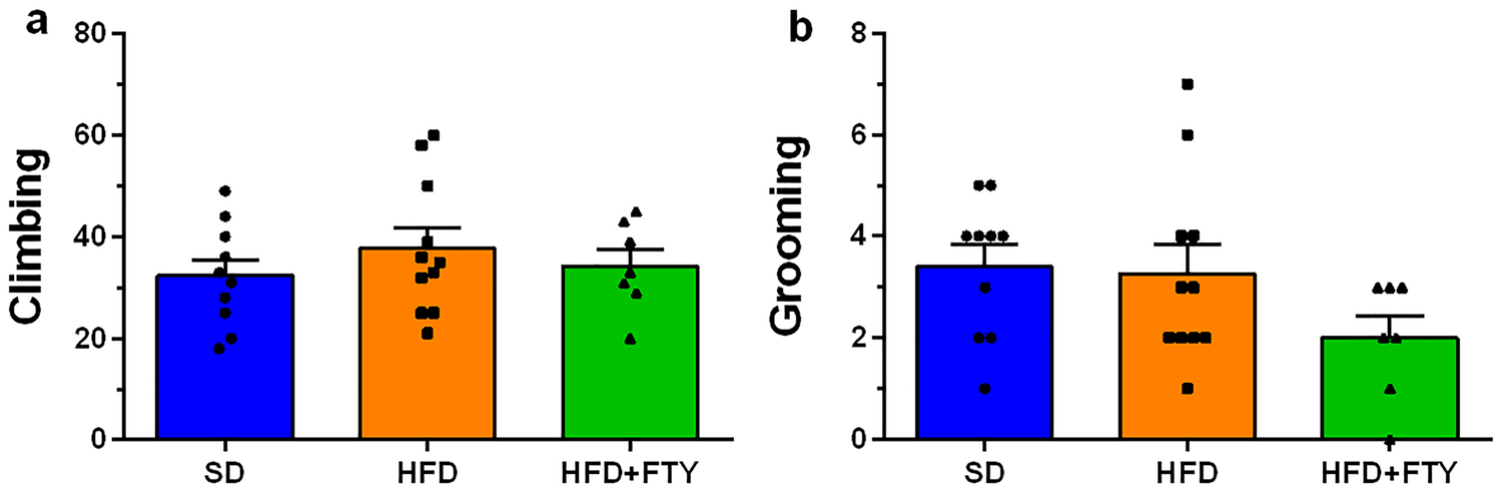
Frequency of exploratory behavior in the OF test: number of climbs (**a**) and grooming behaviors (**b**) in mice that received a standard diet (SD, *n* = 10), mice fed a high-fat diet (HFD, *n* = 11), and animals additionally treated with FTY720 (HFD + FTY, *n* = 7)

The OF test allows quantification of animal exploration and various locomotor parameters, e.g., climbing, grooming, total distance traveled, and velocity. Animals fed a HFD were hyperactive and exhibited increased motor activity, but administration of FTY720 reversed this effect. We also observed an elevation of traveled distance in the OF test in mice receiving a HFD. After simultaneous administration of FTY720, behavioral abnormalities in rodents consuming a HFD were reduced, and the parameters returned to the values observed in animals receiving a standard diet (Fig. 7a). A similar dependence was observed in velocity—the speed of movement was higher in only the group on a HFD (Fig. 7b). Tracking analysis of movement trajectory and heatmaps that represent weighted occupancy across the entire 8-min trial showed that in animals, a HFD caused an increase in the number of entries into the central zone of the apparatus versus other experimental groups (Fig. 8a), but time spent in the central part of the testing box was comparable in all animals (Fig. 8b). Simultaneously, there were no significant differences in the amount of defecation, which indicates a similar level of anxiety in this test among all experimental groups (data not shown). Likewise, there were no significant changes between experimental groups in other parameters of animal activity, such as vertical exploratory behavior, climbing (Fig. 9a), or grooming (Fig. 9b).

#### Novel Object Recognition

In this test, we noticed that a high-fat diet alone and in combination with FTY720 treatment basically did not change the exploratory behavior in mice both during the familiarization and the test phase compared to the animals on a standard diet.

The discrimination index (DI) measures discrimination between novel and familiar objects and is used to evaluate exploratory and recognition memory in the NOR test. Rodents exhibit a natural proclivity for exploring novelty; therefore, animals that remember the familiar object will spend more time exploring the novel object. An increase in the DI—the most relevant parameter in the NOR test—means an improvement in memory retention for the familiar object in animals [38, 39]. In our study, we found no significant difference in the main NOR parameters, such as the DI, the time that mice spent exploring the two identical objects during the familiarization phase, or the time spent exploring the familiar and new objects during the choice phase (*p* > 0.05) (Table 1).

**Table 1:** Main parameters of the NOR test for mice on a standard diet (SD, *n* = 8) and animals on a high-fat diet alone (HFD, *n* = 10) and in combination with FTY treatment (HFD + FTY, *n* = 7)

**Group**	**Familiarization phase—exploration time** **(mean ± SEM)**			**Choice phase—exploration time** **(mean ± SEM)**		
	***t***_**A1**_** (s)**	***t***_**A2**_** (s)**	**DI**_**A1A2**_	***t***_**A1**_** (s)**	***t***_**B**_** (s)**	**DI**_**A1B**_
**SD**	6.36 ± 2.77	8.91 ± 2.90	− 0.33 ± 0.15	9.01 ± 4.76	27.55 ± 3.17	0.63 ± 0.16
**HFD**	11.62 ± 3.24	21.91 ± 6.60	− 0.19 ± 0.22	8.77 ± 2.84	17.15 ± 6.72	0.31 ± 0.12
**HFD + FTY**	11.18 ± 3.88	7.971 ± 1.86	0.01 ± 0.14	8.79 ± 2.48	29.32 ± 7.69	0.51 ± 0.10

*t*_A1_, *t*_A2_, *t*_B_ time spent exploring individual objects, DI_A1A2_, DI_A1B_ discrimination index

In addition, the evaluation of the global time spent examining the objects during the entire test did not show any difference between the groups (Table 2). However, a more in-depth data analysis revealed that the HFD was accompanied by significant reductions in the Recognition Index (RI) performance—the time spent investigating the novel object relative to the total object investigation [30, 33]. In our experiment, animals receiving a SD and HFD combined with fingolimod exhibited novelty preference compared to those on a HFD. It is assumed that RI values greater than 0.50 indicate a novelty preference and thus recognition memory [32, 40].

**Table 2:** Global parameters of the NOR test for mice on a standard diet (SD, *n* = 8) and animals on a high-fat diet alone (HFD, *n* = 10) and in combination with FTY treatment (HFD + FTY, *n* = 7)

**Group**	**Global parameters**		
	***t***_**A1A2**_** + *t***_**A1B**_** (s)**	**GHI**	**RI**
**SD**	51.82 ± 11.33	0.25 ± 0.05	0.63 ± 0.09
**HFD**	59.44 ± 15.15	0.60 ± 0.05^***^	0.25 ± 0.03^**^
**HFD + FTY**	57.26 ± 11.37	0.34 ± 0.07^**##**^	0.49 ± 0.04^**#**^

t_A1A2_, t_A1B_ total time spent in exploration of both objects, GHI global habituation index, RI recognition index

^**^ HFD vs. SD, *p* < 0.01; ^***^HFD vs. SD, *p* < 0.001; ^#^HFD + FTY vs. HFD, *p* < 0.05; ^##^HFD + FTY vs. HFD, *p* < 0.01 (ANOVA with Tukey’s post hoc test)

Mice on a HFD had a significantly lower RI, consistent with their impaired cognitive function. Simultaneous administration of fingolimod caused the recovery of this parameter to correct values, which may indicate its neuroprotective properties. A higher RI reflects good recognition memory, and fingolimod administration ameliorates recognition memory deficits in mice consuming a HFD. There were also differences in the index of global habituation (GHI)—parameters determined by comparing the total time spent exploring the two objects during the familiarization phase to that spent in the test phase. The GHI allows us to determine the overall level of exploration as well as the side and object preferences [33, 41]. A higher GHI usually indicates less interest in the objects in the next trial [42]. The GHI was higher in mice on a HFD, indicating less interest in the novel object. Normalization of both parameters in response to fingolimod administration reflects the therapeutic effect of these drugs in animals fed an HFD.

#### Elevated Plus Maze

**Fig. 10:**
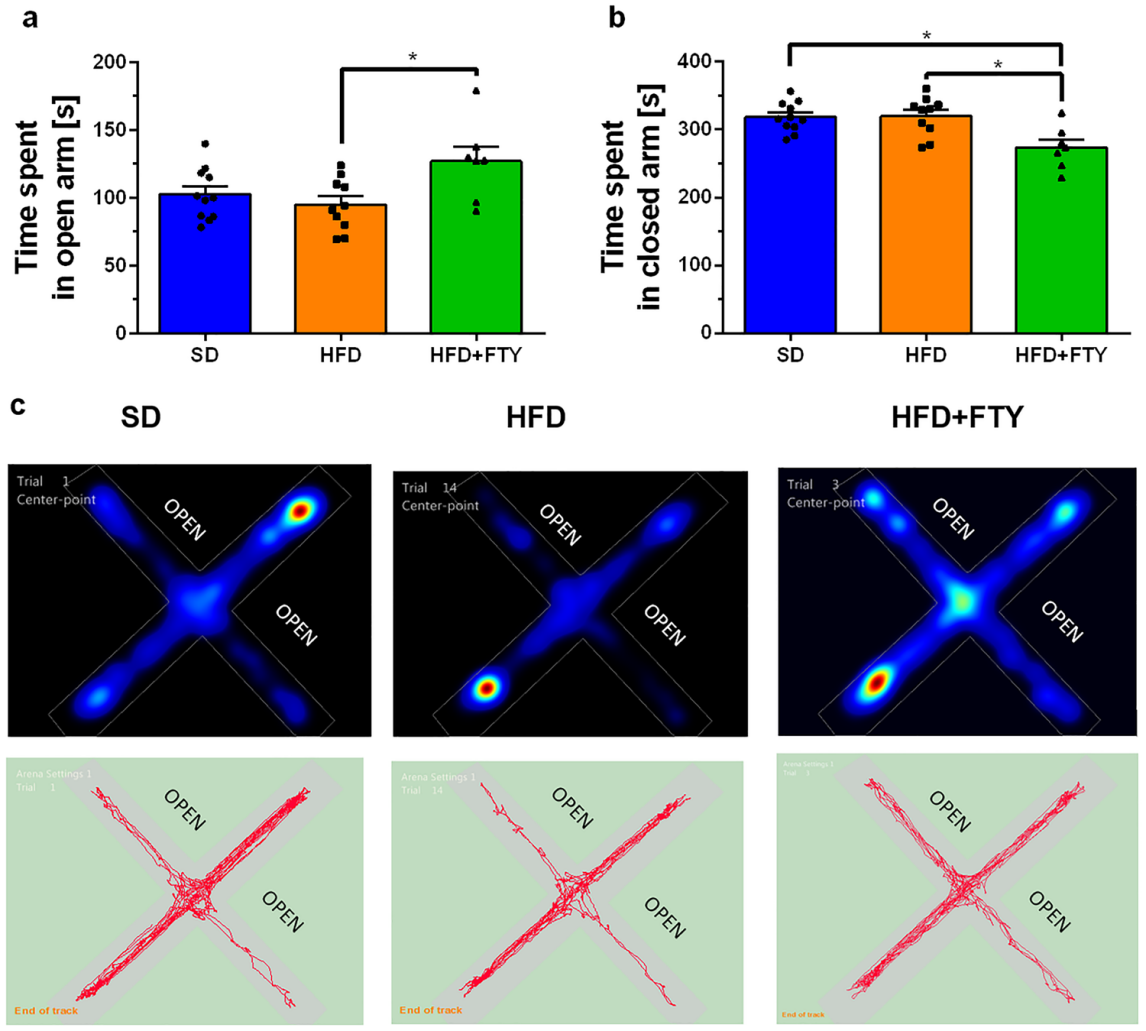
Arm exploration in EPM for the mice that received a standard diet (SD, *n* = 11), mice fed with a high-fat diet (HFD, *n* = 10), and animals treated additionally with FTY720 (HFD + FTY, *n* = 7). Time spent in the open arms (**a**) and closed arms (**b**) of the EPM apparatus. Representative movement trajectory of mice: track plot and heatmaps–occupancy plot (**c**). *HFD + FTY vs. SD, HFD, *p* < 0.05 ( ANOVA with Tukey’s post hoc test)

**Fig. 11:**
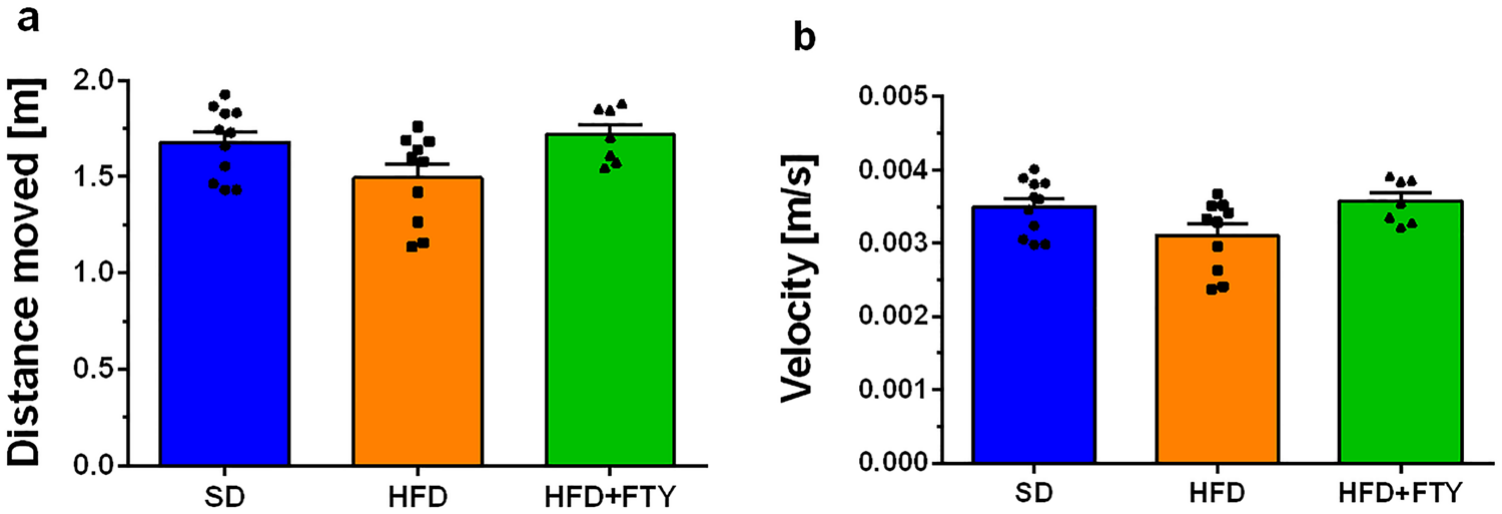
Distance moved (**a**) and velocity (**b**) in the EPM for the mice that received a standard diet (SD, *n* = 11), mice fed with a high-fat diet (HFD, *n* = 10), and animals treated additionally with FTY720 (HFD + FTY, *n* = 7)

FTY720 administration influenced the response on the state-anxiety test. Fingolimod significantly reduced anxiety-related mouse behavior in the elevated plus maze. Statistical and tracking analysis of movement trajectory as well as heatmaps that represent weighted occupancy during the whole test showed that FTY720 mice exhibited decreased anxiety-like behavior when compared to the mice fed with only a HFD and spent more time on the aversive open arms of the apparatus (Fig. 10a). In turn, the SD and HFD groups were more anxious and more likely to stay in safe parts of the maze—closed arms of the apparatus—than animals that received fingolimod (Fig. 10b). Simultaneously, distance moved (Fig. 11a) and velocity (Fig. 11b) were comparable in all animals. The results suggest an anxiolytic effect of fingolimod without significantly affecting motor activity.

## Discussion

Bioactive lipids are key components of cell membranes that control cellular transport and signaling. While the role of ceramide in the development of obesity and T2DM is quite extensively studied, the role of S1P and its receptors is ambiguous, especially when its function depends on location, cell type, and different S1P receptor subtype expression and signaling (i.e., intracellular, extracellular). SPHK1, the key enzyme in S1P metabolism, is involved in various biological processes, including inflammation, atherosclerosis, and angiogenesis. A growing body of research emphasizes the role of SPHK1 in regulating glucose and fat metabolism and suggests its potential use as a promising therapeutic target for obesity and T2DM [43]. Unfortunately, the exact role of SPHKs/S1P in obesity and T2DM is still obscure, and there are many contrasting results from research on animal models. Furthermore, elevated plasma S1P levels are also positively associated with obesity in humans [44]. In the present study, we observed significantly elevated gene expression of *Sphk1* in both the cortex and hippocampus of HFD mice. These results correspond with other studies on HFD animals, where elevated Sphk1 levels were found in other tissues [43, 45]. Mice lacking SPHK1 (SK1^fatKO^) in adipose tissue (AT) after 18 weeks of HFD challenge exhibited impaired glucose tolerance as well as dysregulation of lipolysis and impairment of lipolytic machinery in adipocytes and adipose explants [46]. Other studies have shown that SphK1 overexpression in obese mice promoted hepatic lipid accumulation, whereas its downregulation prevented hepatic steatosis [47]. Furthermore, SPHK1 deficiency in obese mice increased markers of adipogenesis and adiponectin, reduced the expression of proinflammatory cytokines (TNF-α and IL-6), enhanced adipose and muscle insulin signaling, and improved systemic insulin sensitivity and glucose tolerance [48]. On the other hand, KK/Ay diabetic mice injected with SPHK1 revealed the potent hypoglycemic effect of SPHK1 that can improve lipid profiles in type 2 diabetic mice and activate Akt, inactivate GSK3β, and increase glycogen accumulation [49]. Taken together, these data indicate that SPHK1 alteration plays an important role in the regulation of inflammatory processes and glucose homeostasis.

In our study, we mainly focused on S1PR1 and S1PR3, which are primarily expressed in the immune, cardiovascular, and central nervous systems. Knowledge about the function of S1PR3 in the brain is rather unclear, but a recent study revealed that S1PR3 might contribute to the modulation of neuronal excitability and synaptic transmission by S1P. Our work showed that the mRNA levels of *S1pr1* were significantly downregulated in the brains of obese mice in both the cortex and hippocampus, while the gene expression of *S1pr3* remained unaltered. At the same time, a significant increase in *S1pr3* was observed after FTY720 administration in the hippocampus, and a slight increase in gene expression was observed in the cortex of obese mice. The administration of fingolimod did not alter *S1pr1* mRNA levels, although it is being reported as an agonist of S1PR1 and S1PR3. This may be because fingolimod acts as a functional antagonist at S1PR1, internalizing and downregulating the receptor, which was found on lymphocytes [50, 51]. Chakrabarty et al., in a study on HFD mice, suggested that FTY720 appears to be a true agonist of S1PR3 [52]. Transgenic mice lacking S1PR3 showed a significant impairment in appetitively motivated spatial working memory in the T-maze test [53]. The elevation of *S1pr3* mRNA expression after FTY720 observed in the present study corresponds with a previous report, where FTY720 administration to HFD mice significantly upregulated the gene expression of *S1pr3*. Moreover, improved glucose tolerance and insulin-stimulated skeletal muscle glucose uptake and reduced HFD-induced skeletal muscle ceramide accumulation were observed [54]. S1P and its receptors may be involved in the dendritic cell inflammatory response, especially via S1PR3. Niessen et al. revealed that protease-activated receptor 1 (PAR1) signaling amplifies inflammation through SphK1–S1PR3 signaling crosstalk [26]. Moreover, these authors observed that inflammatory exacerbation was attenuated in mice lacking SphK1 after lipopolysaccharide (LPS) challenge. This effect was comparable to that observed in PAR1^−^/^−^ mice and S1PR3^−^/^−^ mice, which showed similar attenuated levels of cytokines. In contrast, Chakrabarty et al. recently revealed that the mRNA levels of S1PR3 are increased in the AT of HFD-induced obese mice, whereas adiposity and glucose homeostasis were impaired in mice deficient in S1PR3, and adipose tissue inflammation and immune cell accumulation were elevated with accompanying hepatic steatosis and inflammation [52]. Moreover, loss of this receptor in combination with a HFD resulted in increased expression of TNF-α and monocyte chemoattractant protein 1 (MCP1), while no changes were observed for IL-6. Interestingly, the expression of S1PR2 was significantly elevated, which may suggest that S1PR2 exacerbates metabolic/inflammatory dysfunction [52, 55]. These observations indicate the importance of a HFD in the induction of the inflammatory response, which may be S1PR3 dependent. Interestingly, Corbett et al. found that S1PR3 mRNA correlates negatively with the severity of posttraumatic stress disorder (PTSD) symptoms and may provide the groundwork for the development of treatment strategies targeting sphingolipid receptors for stress-related psychiatric disorders, including PTSD, anxiety, and depression [56]. The authors found that S1PR3 reduces stress-induced increases in inflammatory cytokines, specifically TNF-α, and regulates medial prefrontal cortex network activity to promote resilience to repeated stress [56]. In the present work, we observed a significant increase in *Il6* and *Tnf* mRNA levels in both the cortex and hippocampus and an increase in *Il1b* in the cortex of obese mice. These results correspond with other studies showing elevated plasma concentrations of lipids and cytokines (including IL-1β, IL-6, IFN-γ, MCP1, and TNF-α) and decreased lipid metabolism-related enzymatic activities in mice fed a HFD for 16 weeks [57]. Our data on HFD mice showed anxiety-like behavior. This may suggest that the upregulation of cytokine expression contributes to obesity-related anxiety-like behavior. TNF-α and IL-1β have been previously shown to induce anxiety-like behavior during the EPM test in rodents [58]. Consistent with our results, Almeida-Suhett et al. found that 16-week HFD-fed mice displayed signs of anxiety-like behavior, and those changes were associated with increased IL-1β levels in different brain regions, but mostly in the amygdala and hippocampus [59]. Mazzoli et al. revealed that there are many similar changes that are present in both AT and key brain areas responsible for learning and memory (frontal cortex and hippocampus) in rats fed a Western diet, including an increase in TNF-α levels and a reduction in adiponectin and brain-derived neurotrophic factor (BDNF) levels [60]. Interestingly, intrahippocampal delivery of an IL1 receptor antagonist prevented synaptic dysfunction, proinflammatory priming, and cognitive impairment in db/db mice [61]. These findings suggest that the use of inhibitors of proinflammatory cytokines might have anxiolytic efficacy. In the present study, we observed a reduction in proinflammatory cytokine mRNA levels in the brains of HFD mice after FTY720 administration. Simultaneously, a significant decrease in anxiety-like behaviors in obese mice after fingolimod treatment was observed.

Since obesity is closely related to T2DM, and Alzheimer’s disease (AD) is also recognized as “type III diabetes,” we checked the expression of genes encoding proteins that are involved in amyloid-beta (Aβ) generation (ADAM10, BACE1, PSEN2, and GSK3β) [62]. Aβ and hyperphosphorylated tau protein are characteristic features of AD. The Aβ peptide is generated in the amyloidogenic pathway via proteolytic cleavage of APP by β- and γ-secretase. Its aggregates are known to have several neurotoxic effects, including oxidative stress, inflammation, and apoptosis. GSK3β is a serine/threonine protein kinase mainly located in the cytoplasm and is known for its involvement in the phosphorylation of tau protein. An emerging body of evidence highly implicates GSK3 signaling in metabolic diseases. GSK3β is also involved in the phosphorylation of AβPP. Blockade of the GSK3β pathway in APP transgenic mice resulted in a reduction in Aβ production and amyloid plaque load [63]. GSK3β may reduce the activity of the α-secretase complex and alter the localization and function of presenilin 1 [64, 65]. In the present work, we observed a significant increase in the mRNA levels of genes encoding BACE1, PSEN2, and GSK3β. Moreover, administration of FTY720 significantly reduced *Bace1* and *Psen2* mRNA levels and downregulated the gene expression of *Gsk3b* in the cortex of obese mice. This may be due to the phosphorylation of Akt and inhibition of GSK3α/β activation, which was previously observed in animals fed a HFD administered FTY720 [66]. A recent study involving a GSK3 inhibitor revealed that GSK3 modulates obesity-induced visceral adipose tissue (VAT) inflammation and that its inhibition has anti-inflammatory effects in an animal model of obesity [67]. In addition, conditional global deletion of GSK3β protected animals from HFD-induced glucose intolerance, but this effect weakened after chronic HFD consumption [68]. The impact of FTY720 on the expression and activity of secretases involved in APP metabolism as well as Aβ levels has been presented in other studies carried out on animal models of AD [69, 70].

Consumption of a HFD induces histone deacetylase (HDAC) activity [71]. We showed significantly decreased hippocampal mRNA levels of sirtuin 1 (SIRT1) in obese mice. SIRT1 is an NAD^+^-dependent protein deacetylase (class III HDAC) that modulates many cellular processes by regulating transcription factors such as FOXO, NF-κB, and p53. Activation of SIRT1 protects HFD mice against negative metabolic consequences by induction of antioxidant proteins and inhibition of proinflammatory cytokines, such as TNF-α and IL-6 via downmodulation of NF-κB activity [72]. The importance of SIRT1 has been recognized in obesity and related disorders, especially when its levels are reduced in white AT of HFD mice because it is cleaved by inflammation-activated caspases [73]. SIRT1 also improves glucose uptake in insulin-resistant adipocytes, promotes lipid metabolism and mitochondrial biogenesis in adipocytes, and coordinates adipogenesis by regulating leptin and adiponectin expression [74, 75]. Moreover, SIRT1 protects against neurodegeneration in AD models by transcriptional activation of the ADAM10 protein, which possesses α-secretase activity on APP [76–78]. SIRT1 also mitigates elevated Aβ levels, Aβ toxicity, and associated inflammatory processes by reducing the NF-κB-mediated transcription of BACE1 [79]. The observed reduction in *Sirt1* gene expression, which was present in HFD mice, may be coresponsible for alterations in pro-inflammatory protein expression and pro-apoptotic Bax encoding genes as well as BACE1, PSEN2, and GSK3β.

In the present study, we also found disturbances in spatially guided exploratory behavior and recognition memory in mice that were fed a HFD during the NOR test, which measures hippocampus-independent memory. We also observed elevated glucose levels in obese animals compared to mice that were fed a standard diet. The obtained results may suggest that impaired glucose levels in obese mice are responsible for memory dysfunction and molecular alterations in proinflammatory cytokines, genes encoding proteins that are involved in APP metabolism (BACE1, PSEN2) and SPHK1, GSK3β, and S1PR gene expression. Moreover, we also observed a reduction in SIRT1 expression in the hippocampus that was accompanied by a decrease in recognition memory. Heyward et al. suggest that obesity induced by a HFD disrupts memory through a mechanism involving the neuroepigenetic dysregulation of Sirt1. The authors indicate that SIRT1 plays a central role in the pathogenesis of obesity-linked memory impairment and that administration of a SIRT1 activator (resveratrol) averts the manifestation of memory impairment in obese mice [80]. Cognitive impairment, represented by reduced place recognition memory that is accompanied by hippocampal upregulation of mRNA levels of IL-1β, IL-6, and TNF-α, has also been recently observed in HFD and fiber-deficient diet mice [81]. Other studies have also shown impairment of memory after 6 weeks of HFD consumption [82]. Similarly, a rapid deficit in spatial recognition memory after 9 weeks, which was followed by an enhancement of anxiety-like behavior after 18 weeks, has been reported in C57BL/6 J male mice that were fed a western diet [83]. In the present study, FTY720 significantly reduced HFD mouse weight, which corresponds to a previous observation where a drug was found to reduce mouse weight gain and additionally reverse insulin resistance and inflammation [21]. Moreover, the administration of FTY720 improved exploratory and recognition memory parameters in obese mice fed a HFD. Similar observations were reported in the rodent model of AD, where administration of fingolimod reduced ceramide levels, inflammation, and Aβ concentration in the cortex of transgenic E4FAD mice and prevented memory impairment [70].

A study by Yoshizaki et al. revealed that 7 weeks of HFD feeding increased hyperlocomotion/exploratory activity and anhedonia-like behavior in the sucrose consumption test [84]. Deng et al. showed that mice fed a HFD for 11 weeks had a marked aggravation of repetitive behaviors (marble burying and self-grooming) and an increased the total distance traveled in the OF test [85]. These results are consistent with our study, as we also observed significantly increased locomotor activity as well as elevated frequency in the central zone of the apparatus during the OF test, which was exclusively present in animals fed a HFD. Another study revealed elevated activity of animals consuming a HFD, but this effect was mostly present during the early period of diet consumption (ca. 2nd week) [86]. Authors suggest that short-term administration of an HFD is significantly associated with behavioral abnormalities but does not impact cognitive impairment. However, other researchers noticed that spontaneous locomotor activity was not affected by a HFD or by a high-fat/high-advanced glycation end-product (AGE) diet [87, 88]. There are also reports showing a reduction in locomotor activity in obese animals [89, 90]. The observed differences in this parameter may be due to different animal ages, species, or the time of HFD exposure. Mifune et al. suggest that diet-induced obesity per se might lead to altered circadian behavioral and molecular rhythms that result in disrupted locomotor activity and increase during the light period and decrease during the dark period [91]. We also did not observe any difference in time spent in the central zone during the OF test, which corresponds to previous results [92], but Gainey et al. revealed that the time spent in the central zone during the OF test might depend on the duration of HFD consumption [82].

No differences in exploration time and time spent in the open arms of the EPM were observed in HFD mice compared to control animals, which corresponds to previous observations [85, 92]. However, Deng et al. noticed that HFD-fed mice entered into the open arms more times than control (nonobese) animals, which suggests that a HFD induces anxiety-like behavior [85]. In our study, the administration of FTY720 significantly increased the time that HFD-fed mice spent exploring the open arms of the EPM. Simultaneously, the drug decreased the time spent in the closed arms. In view of the above, it is reasonably safe to conclude that FTY720 administration decreased anxiety-like behavior in HFD mice in the EPM test. These results correspond with other studies demonstrating that FTY720 exerts anxiolytic and antidepressant-like effects in mice subjected to chronic unpredictable stress. Moreover, S1PR modulator reduced the severity of clinical signs and anxiety-related behavior in experimental autoimmune encephalomyelitis and in a rodent model of AD as well as improved health-related quality of life and depression in patients with relapsing MS [70, 93–96]. Administration of fingolimod even prevents hippocampal memory dysfunction and anxiety-like behavior, the severity of seizures, and related inflammation in rats previously subjected to hypoxia-induced neonatal seizures [97]. A recent study suggests that the reduction in social anxiety- and despair-like behavior in rats after FTY720 treatment may be attributed to reduced mRNA levels of angiopoietin 1, endothelin 1, plasminogen, transforming growth factor beta 2, and matrix metallopeptidase 2 in the medial prefrontal cortex and, consequently, decreased vascular remodeling after drug treatment [98]. Obesity can lead to remodeling of the microvasculature, especially within the hypothalamus, a part of the brain that is involved in the neuroendocrine aspect of stress and anxiety. Furthermore, during HFD feeding, local astrocytes and the vasculature in the hypothalamus undergo profound pathological changes that are not found elsewhere in the brain [99].

**Fig. 12:**
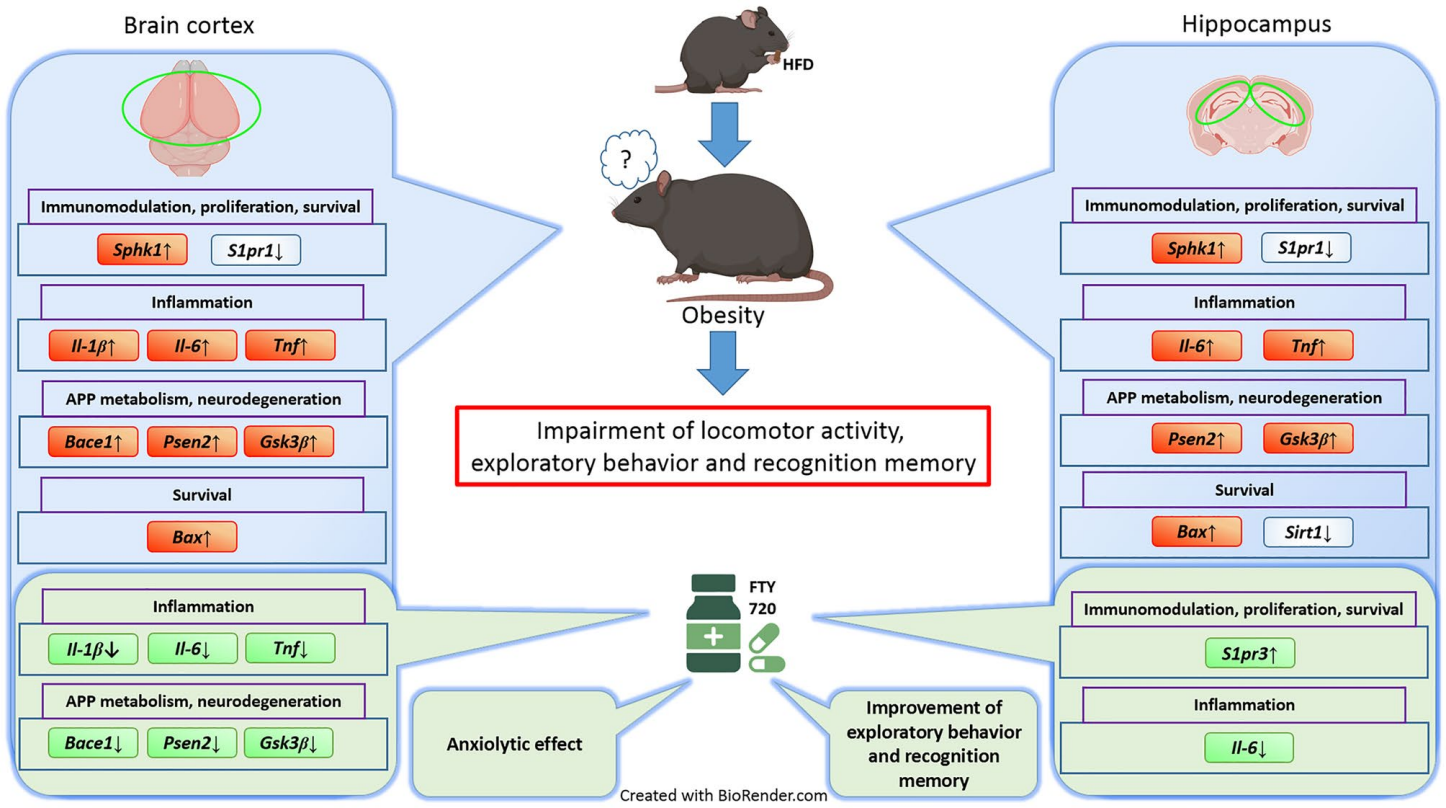
Schematic representation of changes in the transcriptional profile of sphingosine kinase 1, receptors of S1P, inflammatory-related genes, genes encoding BACE1, PSEN2, GSK3β, prosurvival sirtuin 1, and proapoptotic BAX protein in the cortex (left panel) and hippocampus (right panel) of a mouse model of obesity/prediabetes. The effect of fingolimod (bottom green panels) (created with Biorender.com)

In the current work, we have shown that obesity significantly elevated glucose and mRNA levels of *Bace1*, *Psen2*, *Gsk3b*, *Sphk1*, and *Bax* as well as pro-inflammatory *Il1b*, *Il6*, and *Tnf*, which was accompanied by downregulation of *S1pr1* and pro-survival and anti-amyloidogenic *Sirt1*. Moreover, locomotor activity and behavioral parameters reflecting a form of spatial and episodic memory, such as spatially guided exploratory behavior and recognition, were impaired. Administration of FTY720 significantly reduced the weight of HFD animals. Simultaneously, the modulation of S1PRs by fingolimod reversed alterations in the expression of the cytokines, *Bace1*, *Psen2*, and *Gsk3b* that occurred in the brains of obese mice, significantly elevated *S1pr3* mRNA levels, and restored normal behavior patterns as well as cognitive functions; the effect of FTY720 on behavior was still present 4–6 days after administration of the last dose (Fig. 12). Another important action of the drug observed in the study is the anxiolytic effect. Reversal of behavioral and cognitive deficits in this animal model of obesity may suggest a beneficial effect of fingolimod on CNS function.

## Data Availability

The data that support the findings of this study are available from the corresponding author upon request.

## Abbreviations

AD: Alzheimer’s disease
AGE: Advanced glycation end-products
AT: Adipose tissue
Aβ: Amyloid-beta peptide
AUC: Area under curve
Bace1: Beta-secretase 1
Bax: Bcl-2-associated X
BDNF: Brain-derived neurotrophic factor
Cer: Ceramide
CNS: Central nervous system
DI: Discrimination index
EPM: Elevated plus maze test
FTY720: Fingolimod, FTY
GHI: Global habituation index
GSK3β: Glycogen synthase kinase 3β
HDAC: Histone deacetylases
HFD: High-fat diet
IL-1β: Interleukin 1b
IL-6: Interleukin 6
IPGTT: Intraperitoneal glucose tolerance test
MCP1: Monocyte chemoattractant protein 1
MS: Multiple sclerosis
NOR: Novel object recognition test
OF: Open field
PAR1: Protease-activated receptor 1
Psen2: Presenilin 2
PTSD: Post-traumatic stress disorder
RI: Recognition index
S1P: Sphingosine-1-phosphate
S1PR: Sphingosine-1-phosphate receptor
SD: Standard diet
SIRT1: Sirtuin 1
SPHK: Sphingosine kinase
T2DM: Type 2 diabetes mellitus
TNF-α: Tumor necrosis factor α
